# Supplementary material for: Influences of Adult Gender and Parenthood on Adult-Child Interaction Style
Source: Children (Basel). 2022 Nov 24;9(12):1804. doi: 10.3390/children9121804 (PMC9777385; doi:10.3390/children9121804)
Supplement: Supplementary file 1 [file children-09-01804-s001.zip › children-2002603-supplementary.pdf]

Experiences, Perceptions, and Interests Questionnaire (EPIQ)
(Version 1.5)

This questionnaire will ask you about your experiences and perceptions regarding caregiving. Please answer all questions to the best of your knowledge but do not "overthink" your answers. Your first intuition is usually the best answer.

This questionnaire should take you less than 15 minutes to complete. If you have any questions, please ask us at any time or email us at OnlineBabyLab@pitt.edu.

Thank you for your help in our research.

What is your gender?

- ○ Female
- ○ Male
- ○ Other

How many YOUNGER siblings do you have?

- ○ None (0)
- ○ One (1)
- ○ Two (2)
- ○ Three or more (3+)

How many OLDER siblings do you have?

- ○ None (0)
- ○ One (1)
- ○ Two (2)
- ○ Three or more (3+)

Have you ever worked as a babysitter (including for your own siblings)

◯ Yes

◯ No

Have you ever worked as a teacher or teacher's aide?

◯ Yes

◯ No

Have you ever volunteered or worked in a preschool or other childcare business?

◯ Yes

◯ No

On a scale from 0 = no experience to 10 = extensive experience, please rate your own personal experience in interacting with:

Babies (ages 0-2 years)	☆	☆	☆	☆	☆	☆	☆	☆	☆	☆
Preschool children (ages 2-4 years)	☆	☆	☆	☆	☆	☆	☆	☆	☆	☆
Kindergarten children (ages 4-5 years)	☆	☆	☆	☆	☆	☆	☆	☆	☆	☆
Elementary school children	☆	☆	☆	☆	☆	☆	☆	☆	☆	☆
Middle school children	☆	☆	☆	☆	☆	☆	☆	☆	☆	☆
High-school children	☆	☆	☆	☆	☆	☆	☆	☆	☆	☆

Please rate how much you agree with the following statements about yourself:

	Strongly agree	Somewhat agree	Neither agree nor disagree	Somewhat disagree	Strongly disagree
I have a strong feminine side	○	○	○	○	○
I have a strong masculine side	○	○	○	○	○
My closest friends are female	○	○	○	○	○
Most of my friends are males	○	○	○	○	○

How much do you agree with the following statements?

	Strongly agree	Somewhat agree	Neither agree nor disagree	Somewhat disagree	Strongly disagree
1. People can be both aggressive and nurturing regardless of sex	○	○	○	○	○
2. People should be treated the same regardless of their sex	○	○	○	○	○
3. The freedom that children are given should be determined by their age and maturity level and not by their sex	○	○	○	○	○
4. Tasks around the house should be shared equally by men and women	○	○	○	○	○
5. We should stop thinking about whether people are male or female and focus on other characteristics	○	○	○	○	○
6. A father's major responsibility is to provide financially for his children	○	○	○	○	○

7. Men are more sexual than women	○	○	○	○	○
8. Some types of work are just not appropriate for women	○	○	○	○	○
9. Mothers should make most decisions about how children are brought up	○	○	○	○	○
10. Mothers should work only if necessary	○	○	○	○	○

How much do you agree with the following statements?

	Strongly agree	Somewhat agree	Neither agree nor disagree	Somewhat disagree	Strongly disagree
11. Girls should be protected and watched over more than boys	○	○	○	○	○
12. Women rather than men should take maternity leave after giving birth	○	○	○	○	○
13. For many important jobs, it is better to choose men instead of women.	○	○	○	○	○
14. Men are just as effective as women at staying home and taking care of children	○	○	○	○	○
15. When raising children, girls should be taught to be emotional and caring and boys should be taught to be strong and resilient	○	○	○	○	○

16. Because of their biology, mothers are more nurturing than fathers	○	○	○	○	○
17. Being a stay-at-home parent is more fulfilling for women than for men	○	○	○	○	○
18. In times of economic crisis, men should have priority over women to get a job	○	○	○	○	○
19. Female teachers are more effective than male teachers	○	○	○	○	○
20. Children show equal levels of attachment to their mothers and their fathers	○	○	○	○	○

How interested are you in the following activities (0 = less interested, 100 = more interested)

	Less interested	Neutral	More interested
	0		100
Hiking			
Weight training			
Aerobics			
Soccer			
Singing in a Choir			
Making Music			
Cycling			
Dancing			

What race do identify with? (Check all that apply)

- ☐ White
- ☐ Black or African American
- ☐ American Indian or Alaska Native
- ☐ Asian
- ☐ Native Hawaiian or Pacific Islander
- ☐ Other

Are you Hispanic or Latino?

- ◯ Yes
- ◯ No
